# Virtual Simulated Placements in Health Care Education: Scoping Review

**DOI:** 10.2196/58794

**Published:** 2025-06-10

**Authors:** Juliana Samson, Marc Gilbey, Natasha Taylor, Rosie Kneafsey

**Affiliations:** 1 Coventry University, Richard Crossman Building, Priory Street, Coventry, CV1 5FB, United Kingdom, 44 2477659121

**Keywords:** technology, students, learning, scoping review, simulation, healthcare education, virtual simulated placement, practice placement, clinical placement

## Abstract

**Background:**

A virtual simulated placement (VSP) is a computer-based version of a practice placement. COVID-19 drove increased adoption of web-based technology in clinical education. Accordingly, the number of VSP publications increased from 2020. This review determines the scope of this literature to inform future research questions.

**Objective:**

This study aimed to assess the range and types of evidence related to VSPs across the health care professions.

**Methods:**

Studies that focussed on health care students participating in VSPs. Hybrid, augmented reality, and mixed reality placements were excluded. In total, 14 databases were searched, limited to English, and dated from January 1, 2020. Supplementary searches were employed, and an updated search was conducted on July 9, 2023. Themes were synthesized using the PAGER (patterns, advances, gaps, evidence for practice, and research recommendations) framework to highlight patterns, advances, gaps, evidence for practice, and research recommendations.

**Results:**

In total, 28 papers were reviewed. All VSPs were designed in response to pandemic restrictions. Students were primarily from medicine and nursing. Few publications were from low and middle-income countries. There was limited stakeholder involvement in the VSP designs and a lack of robust research designs, consistent outcome measures, conceptual underpinnings, and immersive technologies. Despite this, promising trends for student experience, knowledge, communication, and critical thinking skills using VSPs have emerged.

**Conclusions:**

This review maps the VSP evidence across health care education. Allied health and midwifery research require greater representation, and based on the highlighted gaps, other areas for future research are suggested.

## Introduction

### Background

Practice placements are important activities in the training of health care students. They promote the application of knowledge to a practical setting for developing the skills, attitudes, and behaviors expected of a health care professional [1-3]. Placements allow active involvement in care delivery under supervision, and the opportunity to receive feedback on student performance [4]. In other words, student learning on placement is contextualized to future practice.

Simulation-based placements present an alternative to traditional practice placements. In traditional placements, students enter a workplace and learn through observation and participation in actual clinical events. In contrast, health care simulation is a technique that produces a scenario designed to represent a real-life practice situation for experiential learning [5,6]. Compared with traditional placements, simulation can ensure that low-frequency and high-risk cases or situations receive sufficient practice in a safer space, without mistakes causing harm to real persons [7]. Thus, the advantage of simulation is the ability to control and direct case-based learning.

With advances in technology, simulation-based education is expanding into web-based environments, a trend accelerated during the pandemic. The increasing complexity of health care also requires an agile workforce of lifelong learners, capable of substituting skills across professions [8,9]. Consequently, health care training must keep pace with technology developments, and virtual simulations could support the training of these skills [10-12]. Furthermore, virtual simulations offer greater flexibility and scalability compared with using standardized patients (people play acting the role of a service user) [13].

### Problem Statement

As virtual simulated placements (VSPs) are an emerging field, mapping the literature across health care and analyzing gaps is recommended before more specific research questions are defined [14,15]. Therefore, our research team chose a scoping review method to conduct a broader search across medicine, nursing, midwifery, and allied health, for undergraduate and postgraduate students who undertook VSPs. Considering the importance of practice placement, the advantages of simulation-based learning, and recent advances in technology, this topic was relevant for the review. We define virtual simulations as computer-based activities according to the Healthcare Simulation Dictionary [16], and our aim was to determine the scope of the VSP literature to inform future research questions. Our objective was to assess the range and types of evidence related to VSPs, across the health care professions.

### Review Questions

First, what is the scope of the literature relating to VSPs for health care students? Second, what outcomes are reported in relation to the students undertaking VSPs? Third, what are the patterns and gaps in the literature and the reported outcomes? Finally, what are the implications of the review findings for future directions in VSP research?

## Methods

### Overview

This study followed the stages detailed in a framework for scoping reviews [14]: (1) identify the research question; (2) identify relevant studies; (3) study selection; (4) charting the data; and (5) collating, summarizing, and reporting the results.

A preliminary search of MEDLINE, the Cochrane Database of Systematic Reviews, and Joanna Briggs Institute Evidence Synthesis was conducted on June 17, 2022 to locate any existing or underway reviews on the topic. One systematic review [13] was identified and focused on digital placements for undergraduate nursing and medical students. The review also included experiences such as telemedicine and on-screen role-play. While their search located 16 studies in April 2021, the increased trend toward implementing VSPs within undergraduate and postgraduate programs across the wider health professions justified this review.

An a priori protocol used the Joanna Briggs Institute template for scoping reviews [15] and was registered with the Open Science Framework (DOI 10.17605/OSF.IO/AY5GH) [17]. The PRISMA-ScR (Preferred Reporting Items for Systematic reviews and Meta-Analyses extension for Scoping Reviews) checklist (Checklist 1) ensured methodological rigor when reporting this review [18].

### Relevant Studies

The eligibility criteria are listed in Table 1 using the SPIDER (sample, phenomenon of interest, design, evaluation, research type) [19] and PCC (population, concept, and context) [15] formats:

**Table 1.** Eligibility criteria in population, concept, and context and sample, phenomenon of interest, design, evaluation, research type formats.

Item	Inclusion	Exclusion
S (sample)or population	Papers reporting on undergraduate andpostgraduate health care students, from medicine, nursing, midwifery, and allied health	Papers reporting on professions outside of the target group
PI (phenomenon of interest)or concept and context	Virtual simulation learning in a practice placement.Articles should stipulate that it is a placement, clerkship, elective, selective, practical, or practicum in the curriculum	Onsite simulation Augmented reality and mixed reality interventions Contact with real or standardized patients, even if telecast to students or delivered in a virtual simulation suite Hybrid or blended approaches (part online, part onsite) Tutorials training isolated clinical skills and case studies Theory-based education Assessment of learning
D (design)	Studies with quantitative, qualitative, or mixed methods.	Papers where no research methods were described
E (evaluation)	At least 1 student-centered outcome is included (eg, student satisfaction, confidence, self-efficacy, engagement, learning, knowledge, attitude, skills, or clinical performance)	No student-centered outcomes recorded
R (research type)	Any primary research, including gray literature.In English language and published since January 1, 2020	Reviews—although primary studies will be extracted from relevant reviews to determine their eligibility Study protocols, expert opinion, discussion papers, letters, comments, editorials, and book chapters Survey research (without a virtual simulated placement case)

The selection criteria were piloted by screening 50 randomly selected titles and abstracts, independently by 2 reviewers (JS and MG). This process generated 94% agreement (Cohen κ=0.6) and served to clarify the selection criteria. In discussion with a third reviewer (NT), the Health and Care Professions Council definition for allied health [20] was adopted in place of the National Health Service (NHS) criteria [21], since this definition includes practitioner psychologists—a population potentially well-suited to VSPs, with the emphasis on talking therapies.

### Search Strategy

An initial limited search of MEDLINE and CINAHL was undertaken on June 28, 2022 to identify articles on the topic. The text words contained in the titles and abstracts of relevant articles and index terms were used to develop a full search strategy. This was checked by a health care research librarian and run on MEDLINE on August 3, 2022 (Multimedia Appendix 1). The search strategy was then adapted for each database. The databases searched included MEDLINE, CINAHL, Allied and Complementary Medicine Database, Cochrane Database, PsychINFO, Education Resources Information Center, SCOPUS, ScienceDirect, and Biomed Central. Gray literature sources include PubMed, Electronic Theses Online Service, ProQuest (dissertations), Google Scholar, and Institute of Electrical and Electronics Engineers Xplore. Searches were limited to English language and dated from January 1, 2020. The date limitation was justified given that VSP research has essentially emerged postpandemic.

Supplementary search strategies were employed using existing knowledge and networks, contacting relevant organizations, hand-searching journals, and checking the reference list of all included sources and relevant reviews. Advances in Simulation, British Medical Journal: Simulation and Technology Enhanced Learning (BMJ STEL) and Clinical Simulation in Nursing were hand-searched. These supplementary searches were conducted by one reviewer (JS) and checked by another (NT).

An updated database search was conducted on July 9, 2023. A second reviewer (MG) checked the title, abstract, and full-text selection decisions. Registries (Clinical Trials.gov, World Health Organization International Clinical Trials Registry Platform, and the Cochrane Database) were searched for additional papers [22]. Updated hand searches were performed in Advances in Simulation and Clinical Simulation in Nursing (BMJ STEL had since discontinued). A second reviewer (NT) checked these supplementary searches.

### Source Selection

Following the database searches, all identified citations were uploaded into EndNote (Clarivate) [23], and duplicates were removed. Each potential duplicate was confirmed separately, rather than using batch automation to prevent the removal of false positives [24]. Citations were exported to Rayyan and rechecked for any missed duplicates [25].

Once pilot screening was complete, the remaining titles and abstracts were screened independently by 2 reviewers (JS and MG) against the revised criteria, and potentially relevant sources were retrieved in full text. These were assessed in detail against the inclusion criteria by 2 independent reviewers (JS and MG), blinded in Rayyan. There was 83% agreement (Cohen κ=0.5) between reviewers. A 100% agreement was reached through discussion. Further details of the source selection, including a list of references excluded at full text screening are detailed in Multimedia Appendix 2.

### Data Charting

A Microsoft Excel spreadsheet was used as a data charting tool to standardize obtaining information from the papers. Furthermore, 2 independent reviewers (JS and MG) conducted a pilot of 5 included papers to assess the utility of the information charted and generate emerging themes. Consensus was reached between reviewers (JS and MG) on the charting method, and modifications were made to the spreadsheet, to improve the quality of charted data (Multimedia Appendix 3). Following this, one reviewer (JS) charted the remaining data, which was checked by another (NT).

A table of included study characteristics was collated, and numerical analysis in Microsoft Excel was undertaken to provide descriptive statistics. The size of the dataset was manageable enough to organize findings across the PAGER (patterns, advances, gaps, evidence for practice, and research recommendations) domains [26], for synthesis, without the use of NVivo software (Lumivero; as was planned in the protocol).

### Ethical Considerations

The Coventry University Ethical Approval process has been completed and the project has been confirmed and approved as low risk (project reference P139783). Date of approval is August 12, 2022.

## Results

### Overview

**Figure 1.**
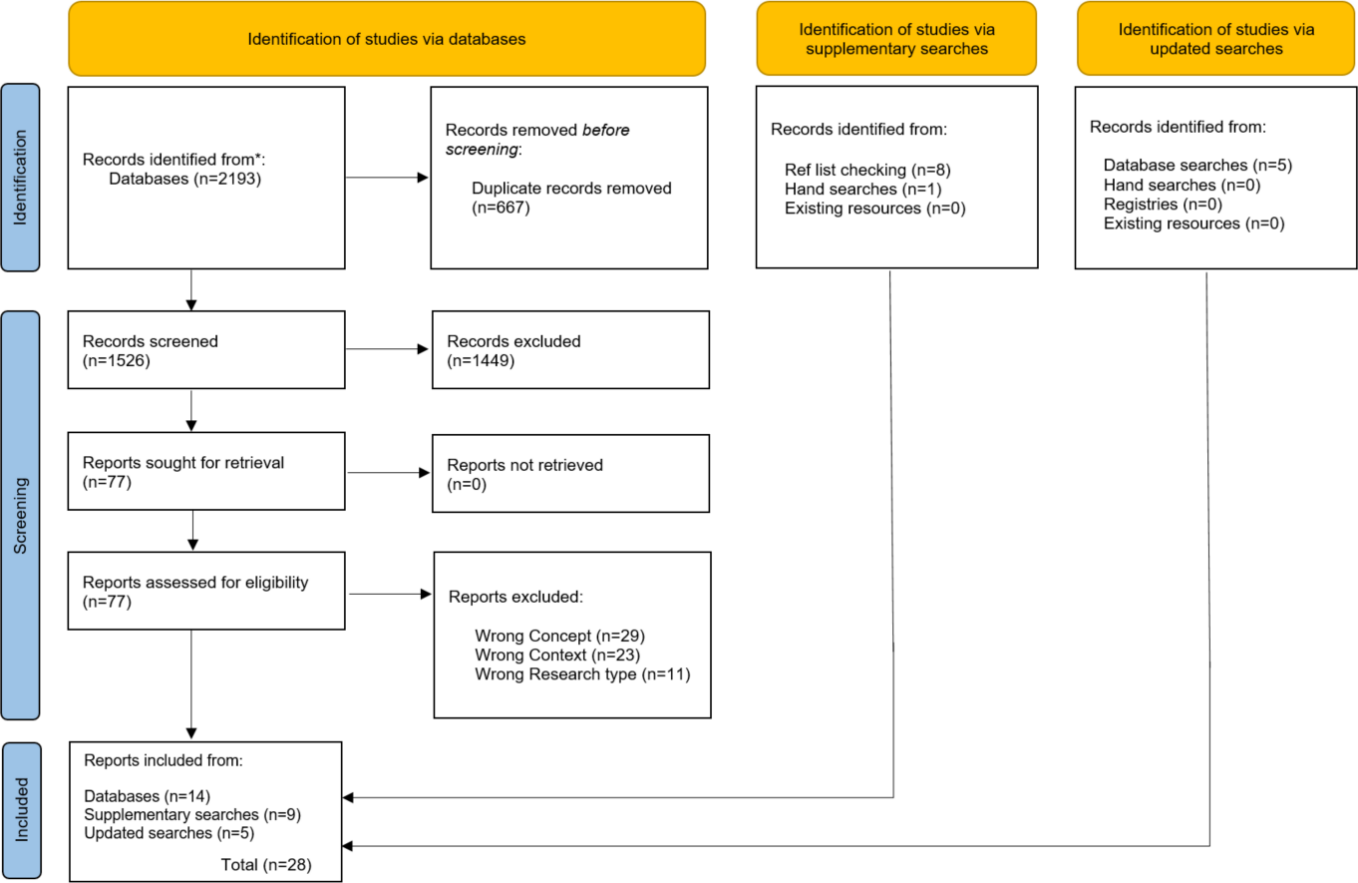
PRISMA (Preferred Reporting Items for Systematic Reviews and Meta-Analyses) flowchart (modified from) [27].

The search results and selection process are reported in the PRISMA (Preferred Reporting Items for Systematic Reviews and Meta-Analyses) flow diagram (Figure 1).

The characteristics of the 28 included papers are summarized in Multimedia Appendix 4. Overall, VSPs were a combination of videoconferencing sessions with educators and peers, as well a variety of web-based material, including videos, reading, modules, and assignments. Most VSPs included some form of case-based learning that required problem-based activities to complete. Teaching methods ranged from didactic lecture-style sessions to peer learning and flipped classrooms. Session delivery featured more formal case conference-style sessions, as well as small group learning, and the use of online chat, polls, and quizzes.

**Figure 2.**
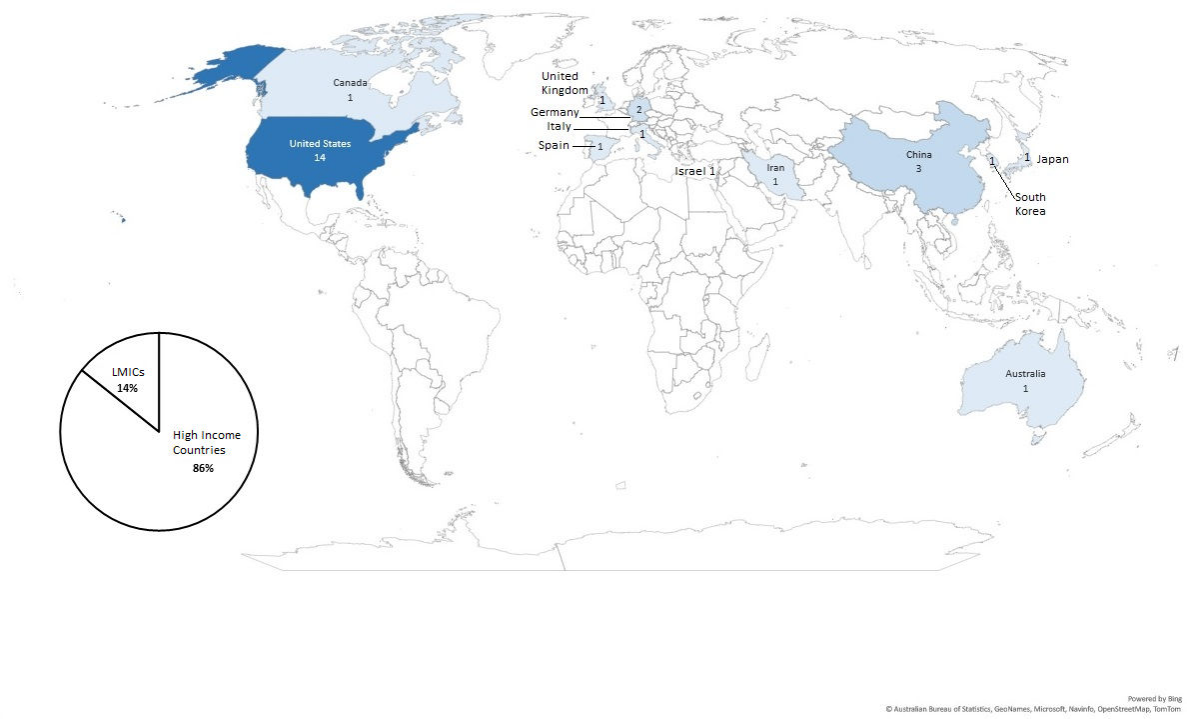
Country of origin of included papers. LMIC: low or middle-income country.

The PAGER themes across the papers are summarized in Table 2. Key patterns and gaps are mapped across all included studies in Tables3  4. The global distribution of publications is illustrated in Figure 2.

Patterns are mapped across all included studies in Table 3 and gaps are mapped in Table 4.

**Table 2.** PAGER (patterns, advances, gaps, evidence for practice, and research recommendations) framework themes summary.

Patterns	Advances	Gaps	Evidence for practice	Research recommendations
Publications from high-income countries (Figure 2)	Innovations occurred mostly in countries with resources to support VSP^a^ development	Few publications from the LMICs^b^	VSPs can be delivered remotely and are scalable (useful for supporting training in the LMICs)	Sharing resources across countries and overcoming barriers such as internet connectivity or access to devices
Narrow profession focus	VSPs occurred within single profession silos. Populations were mostly medical or nursing	No IPE^c^ and minimal allied health representation	Support for VSPs delivering on improved discipline specific skills	The development of IPE VSPs to train skills informed by allied health collaborations
Pandemic response	Rapid innovation to shift from in-person placement to VSPs in response to COVID-19 restrictions	Research planned under time pressure may explain the lack of robust experimental design and conceptual frameworks	Positive outcomes suggest that VSPs could be utilized beyond the pandemic response	With less time pressure, future research could consider conceptual frameworks, with more robust experimental designs
Stakeholder involvement in the VSP design	Most studies involved university faculty. Others also included clinicians	Few incorporated student input and consultation. No evidence of cocreation with service users	Design that involves student participation throughout the process better serves the end user needs	Participatory research designs should include all stakeholders, including students and service users (who ultimately benefit)
Use of generic platforms and screen-based delivery	Platforms such as Microsoft Teams, Zoom, and existing learning management systems were used to facilitate delivery	Limited use of bespoke software or VR^d^. No headsets, haptics, or conversational artificial intelligence systems	Student feedback frequently rated the live interaction with facilitators positively	Bespoke VR software, headsets, and haptic research may emerge as devices become more ubiquitous
A focus on case-based learning	VSPs were oriented toward clinical cases and knowledge, clinical reasoning, decision making, and communication	Practical skills training was rare. Few featured social determinants of health or community interventions	Evidence for improved knowledge, clinical thinking, and communication skills from VSP interventions	Hybrid is currently more suitable for practical skills but haptics may feature as technology improves. Community VSPs link well to IPE
Survey-based outcome measures	Most VSPs were evaluated through custom-designed surveys and student marks	Few validated outcome measure scales or standardized examinations	Evaluations were overall positive and test score improvements were equivalent to in-person cohorts	Validated outcome measures and standardized tests in future trials would provide more robust data for comparison

a VSP: virtual simulated placement.

b LMIC: low or middle-income country.

c IPE: interprofessional education.

d VR: virtual reality.

**Table 3.** Key patterns.

Citation	Patterns			
	High-income country	Medical or nursing profession	Pandemic response	Generic software
Alpert et al [28]	✓	✓	✓	✓
Bhashyam et al [29]	✓	✓	✓	✓
Creagh et al [30]	✓	✓	✓	✓
De Ponti et al [31]	✓	✓	✓	✓
Durfee et al [32]	✓	✓	✓	✓
Fehl et al [33]	✓	✓	✓	✓
Ganji et al [34]	×	×	✓	✓
Gomez et al [35]	✓	✓	✓	✓
He et al [36]	×	✓	✓	✓
Holmberg et al [37]	✓	✓	✓	✓
Joung et al [38]	✓	✓	✓	✓
Kasai et al [39]	✓	✓	✓	✓
Kubin et al [40]	✓	✓	✓	✓
Luo et al [41]	×	✓	✓	✓
Martin-Delgado et al [42]	✓	✓	✓	✓
Nguyen et al [43]	✓	✓	✓	✓
Rahm et al [44]	✓	✓	✓	✓
Redinger et al [45]	✓	✓	✓	✓
Samueli et al [46]	✓	✓	✓	✓
Smith et al [47]	✓	✓	✓	✓
Steehler et al [48]	✓	✓	✓	✓
Taylor et al [49]	✓	×	✓	✓
Villa et al [50]	✓	✓	✓	✓
Weston and Zauche [51]	✓	✓	✓	✓
White et al [52]	✓	✓	✓	✓
Wik et al [53]	✓	✓	✓	✓
Williams et al [54]	✓	✓	✓	✓
Zhou et al [55]	×	✓	✓	✓

**Table 4.** Key gaps.

Citation	Gaps							
	Population		Experimental design				Software	Hardware
	IPE^a^	Allied health	Comparator group	Pre- and postmeasures	Students involved in the design	Conceptual frameworks	Bespoke software	VR^b^ equipment
Alpert et al [28]			✓					
Bhashyam et al [29]						✓		
Creagh et al [30]						✓	✓	
De Ponti et al [31]							✓	
Durfee et al [32]							✓	
Fehl et al [33]			✓			✓		
Ganji et al [34]				✓		✓		
Gomez et al [35]							✓	
He et al [36]								
Holmberg et al [37]				✓	✓			
Joung et al [38]							✓	
Kasai et al [39]				✓		✓		
Kubin et al [40]						✓	✓	
Luo et al [41]				✓		✓	✓	
Martin-Delgado et al [42]								
Nguyen et al [43]						✓		
Rahm et al [44]					✓		✓	
Redinger et al [45]			✓			✓	✓	
Samueli et al [46]						✓	✓	
Smith et al [47]							✓	
Steehler et al [48]				✓	✓			
Taylor et al [49]		✓				✓	✓	
Villa et al [50]				✓	✓	✓	✓	
Weston and Zauche [51]			✓				✓	
White et al [52]						✓	✓	
Wik et al [53]							✓	
Williams et al [54]				✓		✓		
Zhou et al [55]			✓			✓	✓	

a IPE: interprofessional education.

b VR: virtual reality.

### Countries of Origin

In total, 86% (24/28) of the included papers were published in high-income countries, as defined by the Organisation for Economic Co-operation and Development [56]. The VSP research was located primarily in the United States and the Northern Hemisphere.

### Range of Professions

The literature was predominantly medical and nursing research, constituting 93% (26/28) of the included papers. The distribution by profession and breakdowns by specialty are illustrated in Multimedia Appendix 5. Diagnostic radiology rotations were the most prevalent VSPs in medicine and pediatrics in nursing. Where stated, learners were often in their latter stages of training, or undertaking these VSPs as postgraduates.

### Pandemic Response

All the VSPs in the included papers were developed in response to COVID-19 restrictions, which aligns with the time span of the scoping search. The context at the time was that the pandemic necessitated that face-to-face (FTF) practice placements were often discontinued. VSPs were implemented to provide alternative placement hours, enabling students to progress toward professional registration and graduation.

### Experimental Designs

The most basic study design was a single group, with a postintervention measure, featuring in 16 papers. In total, 7 papers compared measures pre- and postintervention [34,37,39,41,48,50,54]. Furthermore, 5 papers compared VSP outcomes with a previous cohort of students who completed FTF placements prepandemic [28,33,45,51,55].

### Stakeholder Involvement

Practice partners (clinicians working in practice) were involved in the VSP course development with faculty in 8 studies [29,32,37,41,44,48,50,55] and students were involved in 4. Furthermore, 3 studies developed a needs assessment from student surveys [34,43,52]. None involved service users.

### Conceptual Frameworks

Conceptual underpinnings include pedagogy, theoretical frameworks, and professional standards. Although no single paper covered all elements, underpinning concepts are evident across the literature, summarized in Multimedia Appendix 6. Pedagogies employed, focused on adult student learners, case-based activities, and experiential and web-based learning. The frameworks structured the VSP development, and the professional standards guided curriculum, simulation, and placement.

### Software

All studies used generic software such as Zoom (Zoom Communications) or Microsoft Teams for screen-based communication, and many used existing learning management systems to host files and activities. Others adopted commercial software applications, allowing students to conduct a history by selecting from a menu of interview questions. None used conversational artificial intelligence (AI) systems (computer-generated conversation, assisted by AI). Some applications presented virtual reality (VR) patient avatars with which the student could direct a physical examination, although this was delivered via a screen [31,38,40,41,51] and 1 study provided an interactive community setting in screen-based VR [53]. All software resources are outlined in Multimedia Appendix 7.

### Intended Learning Outcomes

The focus of most VSPs was clinical cases, through which knowledge, reasoning, decision-making, and communication skills (both verbal and written) were developed. Practical skills training was rarely practiced, with 1 study including home practice surgical kits as the exception [1]. Instead, skill learning was visualized through virtual patient encounters and instructional or walk-through procedure videos. The social determinants of health were the focus in 2 studies [50,53] and another facilitated students in teaching roles [42].

### Outcomes

The most common outcome measures were custom-developed student evaluation questionnaires, followed by exam marks. Custom questionnaires provided positive feedback for student experience, satisfaction, and usability, although some technical issues and Zoom fatigue were cited [31,50]. In total, 3 papers reported a 100% pass rate on their VSPs [35,49,52], and 4 used a standardized exam to demonstrate comparable outcomes with FTF cohorts [45,51], or the national average [30,32].

Table 5 summarizes the outcomes of research that employed a repeated measures design or group comparisons.

**Table 5.** Outcomes from intra- and intergroup comparisons.

Study feature and outcomes	Papers
Measures compared pre- and post-VSP^a^	
Increase in self-rated competencies	Holmberg et al [37], Kasai et al [39], and Williams et al [54]
Increase in knowledge scores	Ganji et al [34], Steehler et al [48], and Villa et al [50]
Improvement in interview skills	Ganji et al [34]
Improvement in critical thinking ability	Luo et al [41]
Comparison between a VSP group and a previous cohort that attended a FTF^b^ placement	
No significant difference in exam scores between groups (*P*>.05)	Redinger et al [45], Weston and Zauche [51], and Zhou et al [55]
Mixed outcomes from survey responses	Fehl et al [33] and Alpert et al [28]

a VSP: virtual simulated placement.

b FTF: face-to-face.

When measures were compared pre- and post-VSP, there was a trend of improvement in self-rated competencies, knowledge scores, and critical thinking skills. However, when the comparison is made with traditional FTF placements, the pattern is less clear. There were no differences in grades when post-VSP exam scores were compared with previous cohorts’ who attended an FTF placement prepandemic. Student satisfaction was comparable in a study conducted in medical general practice, but professional exchange and learning scored higher in the VSP, while the attainment of new skills and attitudes scored higher in the FTF placement [33]. Furthermore, 1 paper compared students who participated in web-based readouts (the radiology equivalent of patient rounds) with students who attended workplace readouts prepandemic [28]. The educational value was comparable in survey results, although students on the VSP rated slightly higher for perceived interaction. That FTF students that were mostly observing on their placement might explain this finding. Conversely, FTF students had greater confidence in using the workstations, considered the case because the VSP students were unable to operate Picture Archiving Communication System workstations remotely.

## Discussion

### Principal Findings

This study mapped the literature describing VSPs across health care. All 28 papers were pandemic responses, primarily from medicine and nursing in high income countries . Selecting studies that conducted a web-based simulation, rather than employing a hybrid or blended approach may explain why all papers in this review were pandemic responses, and why the student populations were in their latter stages of training or postgraduates. COVID-19 necessitated a rapid shift to provide VSPs as a replacement for lost clinical hours to allow students to progress toward graduation [57]. However, these VSPs were often produced in a short time frame, under emergency situations, and may explain why few papers featured robust experimental designs and conceptual frameworks.

Replacing FTF placement hours with simulation is a contentious issue. Accordingly, a Delphi study considered the benefits and limitations of this approach [58]. Expert consensus across multiple professions agreed that between 11%‐30% of hours replaced with simulation would be acceptable, and this aligns with the current allocation set by the Nursing and Midwifery Medical Council [59]. VSPs in the curriculum may offset some pressure on workplace settings as they attempt to fulfill the NHS long-term plan to recruit and train more health care learners [11]. However, this does not diminish the importance of building further workplace placement capacity [58]. VSPs can be considered an additional pedagogy that offers a different, yet complimentary experience to traditional FTF placements.

### Content and Technologies

In general, VSPs had a teleconferencing and a web-based learning component. The teleconferencing was commonly conducted with educators and peers over Zoom or Microsoft Teams, and the web-based learning activities included, but were not limited to videos, reading, modules, and assignments. There were a few examples of immersive learning with VR patient avatars, and these were delivered via a screen [31,38,40,41,51,53].

Disciplines that rely on image-based diagnoses may be more easily adapted to screen-based delivery, and consistent with this, diagnostic radiology, and pathology VSPs together constituted over 30% (6/19) of the medical papers in this review. In the development of this scoping review, we anticipated that psychology might be suited to VSPs due to the nature of talking-based therapies over physical skills, although it is possible that psychological presentations were considered too complex to portray accurately in computer-based simulations. With future developments in conversational AI systems and the growing acceptance of this technology, this situation may change. Similarly, professions that rely heavily on hands-on assessment, such as physiotherapy, may feature more in extended reality spaces with haptics, as further research and development into these technologies emerge. In the meantime, VSPs that require complex conversations are likely to include telecast or telemedicine simulations. Likewise, VSPs that teach advanced handling skills might adopt a hybrid or blended approach, thus combining the strengths of both web-based and FTF approaches.

### Interprofessional Education

VSPs have the potential to break down silos between professions, by delivering interprofessional education (IPE) over a web-based platform. IPE is defined as 2 or more professions, “*learning with from and about one another to improve collaborative practice and quality of care*” [P4] [60]. The intended outcome is to improve mutual understanding, teamwork, and leadership among different professionals [61]. VSPs have advantages over FTF training in building asynchronous activities for flexibility in timetabling and hosting synchronous activities without geographical constraints [62]. Given the relevance of IPE to quality care and the fit with web-based technologies, IPE-VSPs may be an important area for future research.

### VSP Design and Stakeholder Involvement

Elements of thoughtful VSP design are evident across several papers. Frameworks, such as ADDIE (analysis, design, development, implementation, and evaluation), ensure that there is structure to the process and stakeholder needs are met [30]. Existing curricula [54,55], or processes such as Kern’s 6-step model for curricular development could be used [43,45,46,50,52]. If framed within existing standards [40,49], VSPs can align with specified learning outcomes. Principles in pedagogy, such as andragogy [29,30] and web-based learning [33,50], ensure that VSPs build features that engage students with experiential learning [30] and promote problem-solving [29,30,39] and active reflection [49]. The conceptual underpinnings documented across this body of literature could provide a blueprint for best practice in VSP design.

Stakeholder involvement is a key process to inform the design of a VSP. Service users could inform the content, which is especially important in computer-based simulations, yet no service user involvement was documented. Students are the end users of a VSP, yet they were involved in a minority of studies. When students were involved, surveys informed a needs assessment, or they were consulted early in the process. This is a tokenistic approach compared with cocreation, the preferred method of engaging with stakeholders. Cocreation involves a collective effort with all stakeholders to collaborate across the entire design, development, implementation, and testing phases [63]. One noteworthy research report provided an overview of VSP development within a nursing program, which included input from students, service users, and other universities throughout [64]. Their working group comprised of academics, clinicians, a service user, a carer involvement lead, and an education technology lead. Therefore, in addition to underpinning VSP design with the relevant conceptual frameworks (pedagogical principles, theoretical frameworks, and published standards), broad stakeholder cocreation is optimal.

### Research Designs

The pattern of positive student evaluation, improvement from baseline measures post VSP, and equivalence in exam scores, compared with in-person cohorts, appears promising, although, it should be remembered that the objective of a scoping review is to map the literature for patterns and gaps, rather than in-depth appraisal of the quality of the papers.

The findings compare with a systematic review that examined digital clinical education more broadly [13]. Stand-alone digital education was reported to be as effective as conventional learning for knowledge and practice, in nursing and medicine. However, there are some methodological concerns with this systematic review [13]. There was no a priori protocol, and the study lacked a pilot to test the methods. A librarian’s involvement in verifying the search strategy was not reported, gray literature was not searched, and duplicate processes were absent for the study selection and data extraction stages.

There are several barriers to conducting a systematic review of VSPs across health care. First, there is insufficient research across midwifery and allied health [34,49]. Another consideration is that all student evaluations in this scoping review were custom-designed. Therefore, the inconsistency of outcome measures might prevent meaningful comparisons across papers. One study used previously researched scales for clinical thinking ability, academic self-efficacy, and student engagement, which demonstrated good reliability [41]. Some of the exams were standardized [30,32,45,51], but none compared the baseline marks of each group to determine whether there were differences at the outset. In all cases, VSP exam scores were compared with a previous cohort that attended placement FTF prepandemic, or the national average, rather than adopting a prospective design.

It is clear from the paucity of research outside nursing and medicine, the lack of prospective research designs and inconsistent, nonvalidated outcome measures, that research into VSPs is in its infancy. It is tempting to recommend greater consistency of outcome measures and more robust experimental designs to improve the evidence base. However, such approaches may not fit the study of complex educational interventions such as VSPs. More suitable approaches include quasi-experimental, qualitative, and evaluative designs to examine conceptual underpinnings, VSP cocreation, the mechanisms mediating learning responses, and individual case trends over time.

### Strengths and Weaknesses

The strengths of this study relate to the methodology. A structured process for defining search terms was undertaken, and a librarian was consulted for the search strategy. A range of databases were searched across medical and technology specialties. Gray literature sources were searched, and an updated search included trial registries. An a priori protocol was registered, and a subset of data was piloted to determine the declared changes. Duplicate processes in study selection and data charting were employed, and existing guidelines were used to design the protocol, synthesize the findings, and report the paper.

One weakness is that many health care educators may have implemented VSPs without documenting their practices. As such, a scoping review of the literature will always underestimate the scale and depth of innovation in practice. Limiting the search to English language increased the risk of language bias. While the limited number of publications from low or middle income countries could reflect the language limitation, it is also likely that countries with greater resources were better positioned to make the rapid shift to web-based education and publish their research during a global health emergency. Web-based platforms are suited to sharing resources and overcoming geographical constraints to access expertise, and VSPs present an opportunity to address inequality in health care education moving forward.

### Conclusion

This scoping review mapped the VSP evidence across health care, highlighting patterns and gaps in the evidence base. All papers documented pandemic responses, primarily in medicine and nursing in high income countries. There are notable gaps in the midwifery and allied health research. Although emerging trends for VSPs in this review demonstrate some positive outcomes, this review highlights the need for improvements in VSP design. These include cocreation with a wider range of stakeholders and underpinning by pedagogical principles, theoretical frameworks, and published standards. Research into student engagement using VR headsets, haptics, and conversational AI systems in VSPs, are areas for future research, as immersive technologies and their use cases develop. The pandemic has revealed an opportunity to augment placement capacity through VSPs. There is the potential for future VSPs to feature IPE, thus promoting joined-up care in health care graduates. There is also the opportunity for VSPs to improve local and global access to quality clinical education experiences.

## Supplementary material

10.2196/58794 Multimedia Appendix 1 MEDLINE search strategy.

10.2196/58794 Multimedia Appendix 2 Screening Decisions

10.2196/58794 Multimedia Appendix 3 Revised data charting tool.

10.2196/58794 Multimedia Appendix 4 Table of included study characteristics.

10.2196/58794 Multimedia Appendix 5 Papers by professions.

10.2196/58794 Multimedia Appendix 6 Conceptual frameworks.

10.2196/58794 Multimedia Appendix 7 Bespoke health care technology.

10.2196/58794 Checklist 1 PRISMA-ScR (Preferred Reporting Items for Systematic reviews and Meta-Analyses extension for Scoping Reviews) checklist.

## Abbreviations

ADDIE: analysis, design, development, implementation, and evaluation
AI: artificial intelligence
BMJ STEL: British Medical Journal: Simulation and Technology Enhanced Learning
FTF: face-to-face
IPE: interprofessional education
NHS: National Health Service
PAGER: patterns, advances, gaps, evidence for practice, research recommendations
PCC: population, concept, context
PRISMA: Preferred Reporting Items for Systematic reviews and Meta-Analyses
PRISMA-ScR: Preferred Reporting Items for Systematic reviews and Meta-Analyses extension for Scoping Reviews
SPIDER: sample, phenomenon of interest, design, evaluation, research type
VR: virtual reality
VSP: virtual simulated placement
