# Supplementary material for: Virtual Simulated Placements in Health Care Education: Scoping Review
Source: JMIR Med Educ. 2025 Jun 10;11:e58794. doi: 10.2196/58794 (PMC12280114; doi:10.2196/58794)
Supplement: Multimedia Appendix 3 [file mededu-v11-e58794-s003.docx]

### Appendix 4: Revised data charting tool **(pilot revisions in bold)**

| DATA CHARTING |  |  |  |  |
| --- | --- | --- | --- | --- |
| Author(s) |  |  |  |  |
| Year of publication |  |  |  |  |
| Title |  |  |  |  |
| Journal |  |  |  |  |
| Study location |  |  |  |  |
| **Pandemic response** |  |  |  |  |
| Study design **- comparator / control or pre-post measures** |  |  |  |  |
| Aims, objectives and research questions |  |  |  |  |
| Study population(s) |  |  |  |  |
| Sample size (total) |  |  |  |  |
| Number of groups |  | Group 1 | Group 2 | Group 3 |
| Size of each Group |  |  |  |  |
| Intervention | **Scenario** |  |  |  |
| Intervention description | **Activities** |  |  |  |
| Intervention duration |  |  |  |  |
| **Intervention delivery** | **Software** |  |  |  |
| **Intervention delivery** | **Hardware** |  |  |  |
| **Stakeholders in the design** |  |  |  |  |
| Any underpinning concepts / theories **/ standards** |  |  |  |  |
| **Intended learning outcomes (ILOs) / capabilities** |  |  |  |  |
| Methodology |  |  |  |  |
| Student focussed outcome measures |  |  |  |  |
| Student outcomes |  |  |  |  |
| Important **~~results~~ conclusions** |  |  |  |  |
| Sources of funding / conflicts of interest |  |  |  |  |

This is a Multimedia Appendix to a full manuscript published in the J Med Internet Res. For full copyright and citation information see http://dx.doi.org/10.2196/jmir.xxxx
