# Supplementary material for: Virtual Simulated Placements in Health Care Education: Scoping Review
Source: JMIR Med Educ. 2025 Jun 10;11:e58794. doi: 10.2196/58794 (PMC12280114; doi:10.2196/58794)
Supplement: Multimedia Appendix 4 [file mededu-v11-e58794-s004.docx]

| Appendix 5: Table of included study characteristics | | | | | | | | | | | | | |
| --- | --- | --- | --- | --- | --- | --- | --- | --- | --- | --- | --- | --- | --- |
| Citation:  Author^1^  (year)  Country^2^  Publication^3^ | Purpose / aim | Population:  Profession  (specialist rotation)^1^  Sample Size^2^ | Study Design:  Comparison Groups^1^  Pre / post Measures^2^ | VSP Design:  Stakeholders Involved^1^  Pedagogical Framework^2^ | Desired capabilities | Methodology | Intervention:  Scenario(s)^1^  Activities^2^  Duration^3^ | Delivery:  Software^1^  Hardware^2^ | Student focussed outcome measures | Key Findings | Conclusions/ Implications | Funding  / Conflicts of interest |  |
| [1] Alphert et al. (2021)^28^  [2] USA  [3] Academic Radiology | “To assess perceived student engagement & educational value of a new remote clinical radiology learning environment” p113 | [1] 2^nd^ & 3^rd^ year Medical students (on a Radiology rotation)  [2] 83 | [1]  Conventional placement comparator group. Completed the in person course (mostly observing) within 6 months prior to the pandemic (n=36)  [2] post-test measures compared | [1] Not specified  [2] Not mentioned | Describing salient findings of images and working towards a diagnosis | Quantitative questionnaire | [1] Choice of 4 VRO specialties from: abdominal, breast, chest, emergency, musculoskeletal, neuroradiology & paediatric imaging  [2] VROs of curated cases  Online didactic & small group sessions.  [3] 4 weeks | [1] Webex, broadcasting  Picture archiving and communicat-ion systems (PACS) workstations  [2] Screen based | Student experience:  Perceived sense of involvement, technical limitations & educational value of the learning experience | 87.2% & 75% response rates in grp 1 (VRO) & 2 (in person).  Educational value was comparable & interaction ratings were slightly higher in Grp 1: Perceived a more active role = 3.95(0.77), grp 2 =3.41(0.97) p=0.01. Reported less boredom = 1.93(0.47), grp 2 = 2.41(0.8) p=0.007  Confidence using the PACS was higher in grp 2 = 4.04(0.98), grp 1=2.3(1.16) p=0.0001 | Remote clinical radiology education can achieve a similar experience in fewer contact hours.  An advantage is the potential to standardise the education and provide a variety of images  A disadvantage is the lack of a full PACS workstation and socialisation with colleagues | Not Stated |  |
|  |  |  |  |  |  |  |  |  |  |  |  |  |  |

This is a Multimedia Appendix to a full manuscript published in the J Med Internet Res. For full copyright and citation information see http://dx.doi.org/10.2196/jmir.xxxx

| Citation:  Author  (year)^1^  Country^2^  Publication^3^ | Purpose / aim | Population:  Profession  (specialist rotation)^1^  Sample Size^2^ | Study Design:  Comparison group^1^  pre / post measures^2^ | VSP Design:  Stakeholders Involved^1^  Pedagogical framework^2^ | Desired capabilities | Methodology | Intervention:  Scenario(s)^1^  Activities^2^  Duration^3^ | Delivery:  Software^1^  Hardware^2^ | Student focussed outcome measures | Key Findings | Conclusions/ Implications | Funding  / Conflicts of interest |
| --- | --- | --- | --- | --- | --- | --- | --- | --- | --- | --- | --- | --- |
| [1] Bhashyam and Dyer (2020)^29^  [2] USA  [3] Journal of the American Academy of Orthopaedic Surgeons | To create a learning platform to allow PGY-1 residents to develop basic  orthopaedic knowledge & skills for emergency care & progress from basic to more advanced surgical procedures. | [1] Post graduate year one medical residents on an orthopaedic rotation  [2] 12 | [1] Single group  [2] Post-test design | [1] 1 x chief resident  1x program director  2x program coordinators  [2] Problem based learning was encouraged, given the evidence for benefit to adult learners | Cognitive knowledge and skill development | Quantitative questionnaire | [1] Basic skills/hand, sport, trauma & arthroplasty. 11 surgical techniques outlined  [2] Readings, videos, lectures & case "walk throughs." Videoconference demos & informal feedback on surgical skills  [3] 4 weeks | [1] Zoom  A central repository for pre-recorded lectures  [2] Screen based and use of home practice kits for surgical skills | Student satisfaction  Cost (total, start-up and recurring) | 100% were satisfied with the overall experience, module format, take home kits, helping their knowledge base & skill set. 92% felt it improved preparation for the operating room. Total cost per module per student is $1745 | Described a successful conversion of an in person placement to a virtual boot camp. This could be modified according to local policy, use with cadavers and VR when the technology becomes affordable | Not stated |
| [1] Creagh et al. (2021)^30^  [2] USA  [3] Clinical Imaging | “To meet the academic needs of medical students while providing a safe environment during the pandemic.  …To improve the residents' ability to teach.” p420 | [1] 3^rd^ year Medical Students (on a radiology rotation)  [2] 41 | [1] Single group  [2] Post-test design | [1] Not specified  [2] Founded on the principles of andragogy:  Giving greater control over students’ own education.  Emphasis on problem-centred / experiential learning for relevance | Image interpretat-ion  Patient care & safety  Resource utilisation  Insight into the field  Differential diagnosis  Presentation and teaching skills | Quantitative methods to compare test scores and student evaluations | [1] Abdominal & spinal imaging. X-ray interpretation  Women’s health  Interventional radiography.  [2] Reading materials, lectures, modules  American College of Radiography (ACR) e-learning  ‘Hot seat’ sessions  OSCE style sessions  Conferences / tumour board meetings  Grand rounds presentations  [3] 4 weeks | [1] Aquifer (subscription based services)  Zoom  Webex  [2] Screen based | Student performance assessed with the AMSER (or ACR STARS) exam.  Evaluations on the content and structure. | Mean AMSER score was 75% (range 50–96%), matched the national average of 75%, t(40) = −0.14868, p = .88.  Positive feedback on the content, structure, engagement & time efficiency  Improvement in knowledge, leadership skills, search patterns & presenting findings | Student performance was in line with other courses on a national standardised examination.  The approach demonstrates the effectiveness of virtual radiology clerkships as viable alternatives to onsite rotations | Supported by HCA Healthcare  No conflicts of interest |

| Citation:  Author  (year)^1^  Country  Publication^3^ | Purpose / aim | Population:  Profession  (specialist rotation)^1^  Sample Size^2^ | Study Design:  Comparison group^1^  pre / post measures^2^ | VSP Design:  Stakeholders Involved^1^  Pedagogical framework^2^ | Desired capabilities | Methodology | Intervention:  Scenario(s)^1^  Activities^2^  Duration^3^ | Delivery:  Software^1^  Hardware^2^ | Student focussed outcome measures | Key Findings | Conclusions/ Implications | Funding  / Conflicts of interest |
| --- | --- | --- | --- | --- | --- | --- | --- | --- | --- | --- | --- | --- |
| [1] De Ponti et al. (2020)^31^  [2] Italy  [3]BMC Medical Education | “To assess medical students’  perception on fully online training including simulated clinical scenarios during COVID-19 pandemic.” p1 | [1] 6^th^ year Medical students (Medicine & Surgery rotation)  [2] 122 | [1] Single group  [2] Post-test design | [1] Not specified  [2] Not mentioned | Clinical history taking,  Clinical decision making: ordering physical examination, laboratory /imaging tests and interventions | Quantitative questionnaire | [1] 21 simulated cases: 7 Cardiovascular & cerebrovascular cases  6 Trauma cases  2 Pneumonia cases  2 Infective disorder in pregnancy  2 Gastrointestinal surgery cases  1 Nephrological case  1 Hypoglycaemia case  [2] Introduction (to the case & software), virtual patient based training, debriefing  [3] 42 hours (21x 2hr) | [1] Body Interact  Microsoft Teams  [2] Screen based | Student satisfaction  Student feedback questionnaire | 115 (94%) response rate  90% gave a positive evaluation  93% appreciated the format  77% rated the VR realistic for the initial assessment, the diagnostic activity (94%) & treatment options (81%). 84% considered it useful for future hybrid training. 28% had technical issues with online access. | The online training avoided interruption to placements and the majority of participants gave a positive response (although a proportion reported technical difficulties) | None |
| [1] Durfee et al. (2020)^32^  [2] USA  [3] Academic Radiology | “Describe the design and the logistical challenges involved in structuring a virtual radiology clerkship and assess its efficacy.”  p1462 | [1] Medical students in a radiology rotation  [2] 111 | [1] Single group  [2] Post-test design | [1] The clerkship directors from three hospitals  [2] Not mentioned | Patient and safety centred focus  Image utilisation, interpretation and generation of a differential diagnoses | Quantitative student scores and questionnaires | [1] 19 Aquifer modules (no detail of the cases provided)  [2] Large group didactic lectures  Small group homeroom activities: Topic of the day (flipped classroom) and an unknown case conference (readout session) | [1] Aquifer  Zoom  Poll Everywhere  [2] Screen based | Student performance on the AMSER exam  Student feedback | AMSER scores averaged 85% (64-95%): comparable to the in person course.  50% response rate: 100% rated the course overall as good/excellent.  Suggested improvements commonly related to the didactic lectures | The virtual radiology core clerkship was a successful educational experience for medical students. Students enjoyed the small group homerooms, although personal connections were challenging | None stated |

| Citation:  Author  (year)^1^  Country^2^  Publication^3^ | Purpose / aim | Population:  Profession  (specialist rotation)^1^  Sample Size^2^ | Study Design  Comparison group^1^  pre / post measures^2^ | VSP Design:  Stakeholders Involved^1^  Pedagogical framework^2^ | Desired capabilities | Methodology | Intervention:  Scenario(s)^1^  Activities^2^  Duration^3^ | Delivery:  Software^1^  Hardware^2^ | Student focussed outcome measures | Key Findings | Conclusions/ Implications | Funding  / Conflicts of interest |
| --- | --- | --- | --- | --- | --- | --- | --- | --- | --- | --- | --- | --- |
| [1] Fehl et al. (2022)^33^  [2] Germany  [3] Medical Education Online | To provide  students an insight into general practice with its particularities regarding patient clientele, spatial conditions and economic and organisational structure despite the lack of physical presence” p2 | [1] 4^th^ year Medical students (in GP practice)  [2] 192 | [1] Conventional clerkship group (n=277)  [2] Post-test comparison of student evaluations | [1] Not specified  [2] Principles of 'good online teaching'  i.e. clear learning objectives matching the  curriculum, synchronous /asynchronous teacher-student interaction, promotion of  higher-order thinking & communication skills,  encouragement of active & self-directed learning  while promoting timely completion of tasks & effective time management | Higher-order thinking  Communicat-ion skills | Mixed: Surveys generated quantitative & qualitative data which were analysed separately | [1]SOAP cases: gout, acute vertigo, sore throat, hypertension check-up, acute burning on urination, subacute chest pain, geriatric home visit meds review, vaccination, prolonged cough & acute back pain  [2] 10 SOAP cases partly linked with physical examination videos  Videos / materials to learn about general practice.  Visual diagnosis from images  Live video chats with GP teachers  [3] 2 weeks | [1] Student portal  Email  Video chat  Telephone calls (optional)  [2] Screen based  Phone | Working enjoyment Learning gain Practical relevance Insight into GP work Usage behaviours (devices & chosen teaching formats).  Open questions: 'What did they like about the virtual clerkship?'  'What could be improved?' | 51.6% response rate for group 1 & 100% for group 2.  Group 1: 87.9% enjoyed it 89.9% learned a lot, 76.8% gained practical insights  90.9% perceived high practical relevance. 65.6% welcomed this format into future clerkships.  89% laptop usage.  Clinical cases, videos, visual diagnosis and communication with the GP teachers were valued the most.  Students recommended an increase in clinical case content.  Comparison:  The acquisition of new skills & attitudes were rated superior in the conventional clerkship. | Students welcomed the digital clerkship.  The flexible time management, structure & multifaceted learning content were valued.  It rated comparably to face to face (FTF) learning overall, but online was considered better for teaching theoretical rather than practical skills  FTF GP clerkships may benefit from complementing online teaching, in a blended approach. | None |

| Citation:  Author  (year)^1^  Country^2^  Publication^3^ | Purpose / aim | Population:  Profession  (specialist rotation)^1^  Sample Size^2^ | Study Design:  Comparison group^1^  pre / post measures^2^ | VSP Design:  Stakeholders Included^1^  Pedagogical framework^2^ | Desired capabilities | Methodology | Intervention:  Scenario(s)^1^  Activities^2^  Duration^3^ | Delivery:  Software^1^  Hardware^2^ | Student focussed outcome measures | Key Findings | Conclusions/ Implications | Funding  / Conflicts of interest |
| --- | --- | --- | --- | --- | --- | --- | --- | --- | --- | --- | --- | --- |
| [1] Ganji et al. (2022)^34^  [2] Iran  [3] Nurse Education Today | To determine the effect of a virtual gynaecology clinic training programme on the knowledge and clinical skills of midwifery students | [1] Midwifery interns on a Gynaecology rotation  [2] 47 | [1] Single group  [2] Repeated measures design | [1] Research team and midwifery experts (faculty members & senior lecturers). Students were consulted via an educational needs interview  [2] ADDIE model:  (Analysis  Design  Development Implementation Evaluation) | Knowledge  Clinical skills in Interview & history taking  Problem evaluation  Clinical judgement  Problem assessment/management | Reports on the quantitative part of a mixed methods study | [1] 27 cases including genital infections, abnormal bleeding, menopause, ovarian cysts & abnormal smears  [2] Multiple choice  Webinars, Videos  Cases with questions regarding interview, diagnoses & treatment.  [3] Not stated but 2 days were allocated for each of the 4 stages of the cases | [1] Navid –Learning management system (LMS)  WhatsApp  Adobe connect  [2] Screen based | Knowledge  Skills: Modified Mini- CEX - rated over 4 virtual cases.  Student satisfaction on a scale of 1-9 | Knowledge scores pre & post learning were 10.0 ± 1.74  & 13.80 ± 1.43,  p < 0.001.  Post-training scores improved from satisfactory to excellent for  clinical judgment, consultation efficiency & clinical  competence.  Interview scores increased but remained in the satisfactory range. | Training through virtual clinic promoted the knowledge & clinical skills of  midwifery interns.  A virtual clinic may be used in crisis situations & in combination with  teaching under normal circumstances by strengthening the infrastructure & removing barriers. | Financially supported by the university.  No conflict of interest |
| [1] Gomez et al (2021)^35^  [2] USA  [3] Academic Radiology | To rapidly convert to an in-person diagnostic radiology elective to a remote learning experience. | [1] 2^nd^, 3^rd^ & 4^th^ year Medical students (Radiology rotation)  [2] 116 | [1] Single group  [2] Post-test design | [1] Course directors  [2] Not mentioned | Knowledge  Skills in identifying normal anatomy and common pathology | Mixed data in the survey responses | [1] Not specified  [2] Interactive cases. Readouts & Hot seat cases  Quizzes & jeopardy  Website resources  Zoom chats  Q&A  Narrated PowerPoint student submissions  Journal club discussion  [3] 3 weeks | [1] Pacsbin (image library)  Education websites & modules  Zoom  Nearpod PowerPoint  Microsoft &  Google forms Microsoft Teams Blackboard  [2] Screen based | Learner achievement (via completion of quizzes)  Final exam (modified to reflect the altered course content)  Enrolment metrics  Student feedback | 100% pass rate  Largely positive feedback & gratitude for the opportunity to continue learning  Recommended more, small group learning, interactive / reflective content, trainee led teaching & shorter days | The current state of technology makes radiology particularly well suited for distance learning, & with the proper tools and approaches, effective remote radiology instruction can be achieved. | None mentioned |

| Citation:  Author  (year)^1^  Country^2^  Publication^3^ | Purpose / aim | Population:  Profession  (specialist rotation)^1^  Sample Size^2^ | Study Design:  Comparison group^1^  pre / post measures^2^ | VSP Design:  Stakeholders Involved^1^  Pedagogical framework^2^ | Desired capabilities | Methodology | Intervention:  Scenario(s)^1^  Activities^2^  Duration^3^ | Delivery:  Software^1^  Hardware^2^ | Student focussed outcome measures | Key Findings | Conclusions/ Implications | Funding  / Conflicts of interest |
| --- | --- | --- | --- | --- | --- | --- | --- | --- | --- | --- | --- | --- |
| [1] He et al. (2021)^36^  [2] China  [3] Medical education online | To examine the effect of an online neurology course & whether it can cater for interns from  different programs.  Whether group size has an impact. To analyse how it can be refined. | [1] Medical interns (on a neurology rotation)  [2] 92 | [1] Single group  [2] Post-test design with subgroup analysis  by:  Programme enrolment (3 groups)  Intake (6 groups) | [1] Not specified  [2] Not mentioned | Practical skills (New patient admission, physical exam & medical record writing).  Theoretical knowledge | Quantitative | [1] Nervous system, Cardiopulmonary resuscitation & Lumbar puncture  [2] Small private online courses (SPOC). Didactic, flipped classroom & case based learning.  Videos of ward rounds, typical clinical cases & difficult case discussions.  Interactive case discussions  /conferences & reading  [3] 3 weeks | [1] Tencent class (live broadcast platform).  WeChat group  (for shared files)  PowerPoint  [2] Screen based | Final exam scores  Student evaluation | 100% response rate & consistent positive ratings  99% recommended incorporating the course into future programmes.  No difference in test scores between programs (p < 0.05)  Students groups < 15 had a better learning experience (p < 0.05)  Interactive discussions & analysis were rated highest. | The neurology training course was effective and was highly rated by the interns. | Funding from:  Central South University. National Natural Science Foundation of China. Huxiang  High-Level Talent Gathering Project.  No conflicts of interest |
| [1] Holmberg et al. (2021)^37^  [2] USA  [3] Academic Medicine | To deliver essential elements  of the sub-internship virtually and  to address limited teaching faculty  availability | [1] 4^th^ year Medical students on an internal medicine sub-internship  [2] 10 | [1] Single group  [2] Repeated measures design | [1] Clerkship directors, course director, recent graduates of the in-person course (4 senior medical students - near peers)  [2] Not mentioned | Order-writing  Communication  Clinical reasoning  Using medical literature  Admitting a patient, cross overs & handoffs  Independent learning | Mixed data in the survey responses | [1] Not specified  [2] Orientation & debrief. Student presentations  Interactive lectures  Small-group discussions  Case-based faculty led/peer teaching  Role-play  Resident report  [3] 4 weeks | [1] Zoom  [2] Screen based | Student experience  5 self-rated competencies  The  extent to which the course accomplished  its learning objectives | All self-rated competencies demonstrated significant improvement except the describing how to efficiently admit a patient (didn't reach statistical significance).  Open-ended responses indicated initial skepticism, but the course exceeded expectations. | Our findings and our experiences with  the virtual sub-internship suggest that  a virtual sub-internship can be a high quality educational experience | None |

| Citation:  Author  (year)^1^  Country^2^  Publication^3^ | Purpose / aim | Population:  Profession  (specialist rotation)^1^  Sample Size^2^ | Study Design:  Comparison groups^1^  pre / post measures^2^ | VSP Design:  Stakeholders Involved^1^  Pedagogical Framework^2^ | Desired capabilities | Methodology | Intervention:  Scenario(s)^1^  Activities^2^  Duration^3^ | Delivery:  Software^1^  Hardware^2^ | Student focussed outcome measures | Key Findings | Conclusions/ Implications | Funding  / Conflicts of interest |
| --- | --- | --- | --- | --- | --- | --- | --- | --- | --- | --- | --- | --- |
| [1] Joung and Kang (2022)^38^  [2] South Korea  [3] Issues in Mental Health Nursing | Investigate the potential of VS-based  education as an alternative for clinical  psychiatric nurse training & consider how it can be optimised as an educational method | [1] 4^th^ year Nursing Students (Psychiatry rotation)  [2] 20 | [1] Single group  [2] Post-test design | [1] Not specified  [2] Not mentioned | The transfer of intrinsic  nursing values such as empathy. | Qualitative | [1] Schizophrenia, bipolar disorder, anxiety disorder and depressive disorder  [2] vSim sessions, team conferences with an instructor  [3] 90 hours over 10 days | [1] vSim for Nursing  Video conferencing software  [2] Screen based | Focus Groups | 3 key themes:  1. Students were glad that the patients were not real people  2. vSim serving as a bridge between the text & real world  3. Supplementat-ions needed for vSims to replace clinical practice | vSim was recognised as a tool linking theory with actual clinical practical training.  Students were able to repeat practice to solve problems and work in a safe environment but were unable to have real human experiences. | No funding or conflict of interest |
| [1] Kasai et al. (2021)^39^  [2] Japan  [3] BMC Medical Education | To evaluate the feasibility & effectiveness of this approach. To identify the advantages & disadvantages of online-simulated clinical placement (sCP) from the medical students’ perspectives | [1] 5^th^ year Medical students on a respiratory unit & general medicine rotation  [2] 43 | [1] Single group  [2] Repeated measures design | [1] Not specified  [2] Peer assisted learning applied to problem based learning | History taking  Diagnosis  Select tests & interpret results  Treatment planning  Medical documentation & present the clinical course  Perform safe, evidence based treatment  Informed consent & patient education | Mixed | [1] General medicine outpatient cases & respiratory inpatient cases  [2] Simulated electronic & health records (sEHR)  electronic-Practice Based Learning (e-PBL)  Online virtual medical interviews (VMI)  [3] 4 weeks | [1] Video conference system  Learning management system (LMS)  Microsoft excel  [2] Screen based | Self-evaluation of clinical performance  Semi structured focus groups | 100% response rate  Online sCP is an efficient learning method & useful for learning how to write medical records & summaries  Clinical clerkship was more useful for learning associated with medical interviews, physical examination & humanistic qualities eg. professionalism | Online-sCP with sEHR, e-PBL, and online-VMI could be  useful in learning some of the clinical skills acquired  through clinical clerkship. | None |

| Citation  Author  (year)^1^  Country^2^  Publication^3^ | Purpose / aim | Population:  Profession  (specialist rotation)^1^  Sample Size^2^ | Study Design:  Comparison group^1^  pre / post measures^2^ | VSP Design:  Stakeholders Involved^1^  Pedagogical Frameworks^2^ | Desired capabilities | Methodology | Intervention:  Scenario(s)^1^  Activities^2^  Duration^3^ | Delivery:  Software^1^  Hardware^2^ | Student focussed outcome measures | Key Findings | Conclusions/ Implications | Funding  / Conflicts of interest |
| --- | --- | --- | --- | --- | --- | --- | --- | --- | --- | --- | --- | --- |
| [1] Kubin et al. (2021)^40^  [2] USA  [3] Journal of Nursing Education | To develop an innovative re vised plan for facilitation of clinical learning experiences in the virtual learning environment. | [1] Nursing students on a Paediatric Rotation  [2] Not stated (taught in small groups of 5-10) | [1] Single group  [2] Post-test design | [1] Not specified  [2] International Nursing Association of Clinical Simulation and Learning  (INACSL) best practice guidelines | Nursing process  Growth and development  Assessment  Clinical judgment & reasoning skills  Prioritisation & delegation  Communication skills. | Mixed: Survey with Likert & open ended questions | [1] Various paediatric disorders. Child with diabetic ketoacidosis was used in the escape room  [2] Virtual escape rooms, unfolding video case studies, & blended prioritisation simulations.  [3] Not stated | [1] vSim  NurseThink vClinical  F.A. Davis' Paediatric Interactive Clinical Scenarios  Virtual Healthcare Experience  Flipgrid  Google Forms & Sites  [2] Screen based | Student satisfaction.  Evaluations pf each clinical activity and the ability to meet course outcomes | 100% response rate  Self-reported increases in clinical reasoning, prioritisation, communication and critical thinking skills. | Virtual activities can be as effective as in-person clinical learning methodologies. Integrating virtual activities into clinical curricula can be a viable option, especially in areas where clinical placement is limited | Not stated |
| [1] Luo et al. (2021)^41^  [2] China  [3] Clinical Simulation in Nursing | To understand students’ performance, learning effectiveness & satisfaction with  their participation in distance learning.  To compare outcomes between genders | [1] 4^th^ year Nursing students  [2] 35 | [1] Single group  [2] Repeated measures design | [1] Nursing educators from the University  [2] Outcome- Based Educational theory, which emphasises student centred learning  National Standards for Nursing Undergraduates | Pass the 2020 Chinese Registered Nurse Licensure Exam  knowledge and clinical competence requirements specified in the National Standards | Quantitative | [1] Medical, surgical, obstetrics & gynaecology, paediatrics, fundamental nursing  [2] Webinars (lectures & case based learning) & Virtual simulations  [3] 3 months | [1]Videoconferencing platforms (Tencent Meeting & Ding Talk)  vSim  [2] Screen based | Theoretical knowledge  Clinical thinking ability  Academic self-efficacy  Student satisfaction | 100% response rate  High levels of student engagement, satisfaction & theoretical knowledge.  Significant improvements in Systematic, Evidence based &  Clinical thinking  Females outperformed males in all domains | Distance learning combining webinars & virtual simulations could meet the learning requirements of senior nursing students in a safe environment in a flexible manner, & students could obtain theoretical knowledge & grow their clinical thinking ability | Funded by Wuhan University Teaching & Research reform Project.  No conflicts of interest |

| Citation:  Author  (year)^1^  Country^2^  Publication^3^ | Purpose / aim | Population:  Profession  (specialist rotation)^1^  Sample Size^2^ | Study Design:  Comparison groups^1^  pre / post measures^2^ | VSP Design:  Stakeholders Involved^1^  Pedagogical Framework^2^ | Desired capabilities | Methodology | Intervention:  Scenario(s)^1^  Activities^2^  Duration^3^ | Delivery:  Software^1^  Hardware^2^ | Student focussed outcome measures | Key Findings | Conclusions/ Implications | Funding  / Conflicts of interest |
| --- | --- | --- | --- | --- | --- | --- | --- | --- | --- | --- | --- | --- |
| [1] Martin-Delgado et al (2022)^42^  [2] Spain  [3] Journal of Professional Nursing | To explore final-year nursing experiences from completing their  clinical training in a teaching role practicum during the pandemic. | [1] Final year Nursing students  [2] 34 | [1] Single group  [2] Post-test design | [1] Not specified  [2] Not stated | Designing and developing evidence based educational materials aimed at meeting the learning needs of their peers. | Qualitative | [1] Covid 19 educational needs, including management of respiratory patients, mechanical ventilation, use of protection equipment  [2] Online training Mentoring sessions Design and development of educational material  [3] 3 months | [1] Video conferencing software, Moodle (LMS)  [2] Screen based | Themes from student reflective journals (18 of the 34 students) | Three themes  1. Emotions due to not being able to complete their final placement & not to joining the workforce  2. Perceived benefits of a teaching role,  3. Recognising the teaching role as key to the profession & the  importance of scientific evidence in clinical practice. | The online teaching practicum gave students the opportunity to develop education competencies. | No mention of conflicts of interest. No external funding |
| [1] Nguyen et al (2023)^43^  [2] USA  [3] MedEdPortal | To transition an intro-ductory anesthesi-ology clerkship to an entirely virtual curriculum | [1] 3rd & 4th year Medical students (anaesthesi-ology)  [2] 28 | [1] Single group  [2] Post-test design | [1] Not stated, but past student surveys from placements were used for a needs assessment  [2] Kerns 6 steps of curriculum development | Nine educational objectives were outlined, including information/description, differential diagnosis, treatment prioritisation and planning | Mixed | [1] Preoperative evaluation, inhaled intravenous anaesthetics, airway management, anaphylaxis, malignant hyperthermia and unanticipated difficult airway  [2] Didactics, assigned readings, case based learning discussions  [3] 2 weeks | [1] Canvas (LMS) PowerPoint Simulation videos  Zoom  [2] Screen based | Survey with Likert and open-text responses | 79% response rate. Clerkship met / exceeded expectations in all areas. All students agreed / strongly agreed that the objectives were clear & achieved. Two students indicated that the assessment tools could align better with the curriculum & one wanted more didactics. One noted technical issues with Zoom | A compelling clerkship was executed, which was highly rated. | No disclosures or funding to report |

| Citation  Author  (year)^1^  Country^2^  Publication^3^ | Purpose / aim | Population:  Profession  (specialist rotation)^1^  Sample Size^2^ | Study Design:  Comparison group^1^  pre / post measures^2^ | VSP Design:  Stakeholders Involved^1^  Pedagogical framework^2^ | Desired capabilities | Methodology | Intervention:  Scenario(s)^1^  Activities^2^  Duration^3^ | Delivery:  Software^1^  Hardware^2^ | Student focussed outcome measures | Key Findings | Conclusions/ Implications | Funding  / Conflicts of interest |
| --- | --- | --- | --- | --- | --- | --- | --- | --- | --- | --- | --- | --- |
| [1] Rahm et al. (2021)^44^  [2] Germany  [3] PLOS One | To enrich our understanding of how students perceive realistic multimodal  game-like e-learning cases within a complete e-learning-based curriculum. | [1] Medical students in an internal medicine rotation  [2] 198 | [1] Single group  [2] Post-test design | [1] Medical students (who had already completed the internal medicine module) and physicians from different disciplines  [2] Not stated | Decision-making skills  Communication  Diagnostic thinking | Mixed:  Quantitative survey with free text space for student feedback | [1] Cases based on routine encounters across different clinical settings  [2] e-learning cases with quizzes & interaction modules with gamification  [3] 10 weeks | [1] articulate.com (bespoke creator tool)  Moodle LMS  [2] Screen based | Student evaluation | 49.5 to 82.5% response rates to case evaluations & 25.8% end of term response  Clinical context, interactivity, game-like interface & embedded learning in the cases motivated students to engage with the learning materials & cases | Solving and interpreting e-learning cases close to real-life settings promoted students’ motivation during the COVID-19 pandemic and may partially have compensated for missing  bedside teaching opportunities. | Funded by clinician-scientist-program of the German Internal  Medicine Society (DGIM).  No conflicts of interest |
| [1] Redinger and Greene (2021)^45^  [2] USA  [3] Western Journal of Emergency Medicine | To describe the development, application & program evaluation of a virtual advanced emergency medicine (EM) curriculum developed rapidly in response to the COVID-19 pandemic. | [1] 4^th^ year Medical students on an EM rotation  [2] 104 | [1] Traditional rotation from a previous cohort  (n= 48)  [2] Post-test exam scores compared between groups | [1] Not Specified  [2] Kerns 6 step model for curriculum development | History, physical, diagnosis & case presentation  Common diagnostic studies,  Management plans,  Knowledge, indications / constraints & basic procedural skills.  Emergency recognition & management | Mixed methods | [1] 12 most common chief complaints in clinic  [2] Case series, radiology & ECG interpretation, textbooks, journal articles, podcasts, online board review, blog posts quizzes & a case presentation.  [3] 4 weeks | [1] Microsoft Teams  MedEd Case X  EM: RAP C3 series  SAEM EM Curriculum  Sublux Radiology App  A Night in the ER App  [2] Screen based | Student performance (National Standardised EM Shelf Exam) – simplified to pass/fail  Course evaluation (focus group) | No difference between performance scores t(102) = 1.317  p = 0.174  Comments indicate that the virtual clerkship successfully met their learning needs, resulting from its design, organisation & use of quality learning resources. | Students demonstrated the same levels of knowledge in the virtual & traditional rotations  Feedback was overall positive, although limited peer interaction & group learning dynamics were noted. | None |

| Citation:  Author  (year)^1^  Country^2^  Publication^3^ | Purpose / aim | Population:  Profession  (specialist rotation)^1^  Sample Size^2^ | Study Design:  Comparison groups^1^  pre / post measures^2^ | VSP Design  Stakeholders Involved^1^  Pedagogical Frameworks^2^ | Desired capabilities | Methodology | Intervention:  Scenario(s)^1^  Activities^2^  Duration^3^ | Delivery:  Software^1^  Hardware^2^ | Student focussed outcome measures | Key Findings | Conclusions/ Implications | Funding  / Conflicts of interest |
| --- | --- | --- | --- | --- | --- | --- | --- | --- | --- | --- | --- | --- |
| [1] Samueli et al. (2020)^46^  [2] Israel  [3] Annals of Diagnostic Pathology | To review a diagnostic pathology selective for undergrad medical  Students. Including the design, operation, evaluation, & suggestions for further adjustments. | [1] 3^rd^ and 4^th^ year medical students (diagnostic pathology selective)  [2] 59 | [1] Single group  [2] Post-test measures | [1] Course coordinator  [2] Kerns 6 step framework for curriculum development | Introduce surgical pathology  Reinforce the pathological basis for disease, including mechanisms & treatments  Appreciate “the way a pathologist thinks,” & what they “mean” in their reports, as well as the significance of commonly described findings | Quantitative Survey (with open text options) | [1] Principles of non-neoplastic (inflammatory) & neoplastic (benign/malignant) disorders, Dermatopathology, Breast pathology, Neoplastic neuropathology, Neoplastic thyroid pathology Advanced topics in diagnostic pathology (NUT carcinoma, thyroid pathology)  [2] Self-assigned reading , lectures, slide reviews, diagnostic quiz  [3] 2 weeks | [1] Zoom  PowerPoint Moodle  Whole slide image (WSI) viewers:  (CaseViewer & Aperio ImageScope)  Library subscription (for assigned texts)  [2] Screen based | Student experience (previous exposure to pathology),  Level of interest & learning from the course,  Evaluation / feedback | 42% survey response rate, Participants new to diagnostic pathology instruction. Overall, the course was rated very favourably: 68% gave at least 3 out of 4 points for questions related to course interest, improved understanding of diseases & how strongly they would recommend the course. The key disadvantage as reported by 80% was tech issues accessing the slides | The course was a success and can be a model for future virtual pathology electives.  Great effort should made to provide technical support to the students.  The selective demonstrated value for students and provided much-needed exposure to diagnostic pathology in clinical practice. | None |
|  |  |  |  |  |  |  |  |  |  |  |  |  |

| Citation:  Author  (year)^1^  Country^2^  Publication^3^ | Purpose / aim | Population:  Profession  (specialist rotation)^1^  Sample Size^2^ | Study Design:  Comparison groups^1^  pre / post measures^2^ | VSP Design:  Stakeholders Involved^1^  Pedagogical Framework^2^ | Desired capabilities | Methodology | Intervention:  Scenario(s)^1^  Activities^2^  Duration^3^ | Delivery:  Software^1^  Hardware^2^ | Student focussed outcome measures | Key Findings | Conclusions/ Implications | Funding  / Conflicts of interest |
| --- | --- | --- | --- | --- | --- | --- | --- | --- | --- | --- | --- | --- |
| [1] Smith and Jones (2023)^47^  [2] Australia  [3] BMC Medical Education | Provide an elective that enables students to make the current clinical world relevant, cover key content to assure intern preparedness & explore how COVID-19 changed one key area of medical practice. | [1] 4^th^ year Medical students  [2] 250 | [1] Single group  [2] Post-test design | [1] Academics and professional support (clinicians)  [2] Not mentioned | Clinical communication  To author case studies of COVID approaches | Mixed | [1] Atrial fibrillation, depression, hypertension, prone ventilation, using protective equipment, lung ultrasound. COVID Global health, public health, child health, aged care, legal & ethical, general practice. Primary care, mental health & evidence-based practice.  [2] Podcasts, case studies, flipped classrooms, tutorials & modules  [3] 6 weeks (200 hrs) | [1] Microsoft Teams  PowerPoint  OSLER (logging of progress & assessment) National Prescribing Service (NPS) modules  COVID online modules.  [2] Screen based | Evaluation survey with Likert responses and open-ended questions | 32% response rate. Overall worked well, was well coordinated & a good option for a disrupted placement. The project options met their needs very well & were well supervised. More guidance asked for on COVID information & academic writing support for publication. Some found the OSLER and NPS modules a bit dry. | The COVID-19 e-elective was successful in meeting student learning needs & alleviated the concerns of students whose placements were disrupted. | No external funding and no competing interests |
| [1] Steehler et al. (2021)^48^  [2] USA  [3] Otolaryngology - Head and Neck Surgery | To develop and evaluate a virtual otolaryngology elective created during COVID-19.  To teach the basics of otolaryngology & increase exposure to the specialty | [1] 3^rd^ & 4^th^ Medical students (head & neck surgery rotation)  [2] 12 | [1] Single group  [2] Repeated measures design  (for n=5 pre & post knowledge test scores) | [1] Faculty, residents and senior medical students  [2] Not mentioned | Pathophys-iology  Workup  Differential diagnosis  Treatment of disease course  otolaryngology practice & referral | Mixed methods | [1] Rhinology, otology, facial plastic & reconstructive, laryngology, paediatric otolaryngology, imaging, & emergencies.  [2] Orientation, anatomy/examination & surgical videos, reading, lectures, case based learning, grand rounds & roundtable conversation  [3] 1 week | [1] Zoom  [2] Screen based | Test scores  (pre & post for n=5)  Student evaluation | 92% reported increased understanding & interest in the field  Increase in knowledge test scores (p=0.001).  Appreciation for course organisation, formative assessment & case based learning | An virtual otolaryngology elective format can be effective at providing an educational experience & garnering interest | None |

| Citation:  Author  (year)^1^  Country^2^  Publication^3^ | Purpose / aim | Population:  Profession  (specialist rotation)^1^  Sample Size^2^ | Study Design:  Comparison group^1^  pre / post measures^2^ | VSP Design:  Stakeholders Involved^1^  Pedagogical frameworks^2^ | Desired capabilities | Methodology | Intervention:  Scenario(s)^1^  Activities^2^  Duration^3^ | Delivery:  Software^1^  Hardware^2^ | Student focussed outcome measures | Key Findings | Conclusions/ Implications | Funding  / Conflicts of interest |
| --- | --- | --- | --- | --- | --- | --- | --- | --- | --- | --- | --- | --- |
| [1] Taylor et al. (2021)^49^  [2] UK  [3] British Journal of Nursing | To explore & discuss a simulated clinical placement, aimed at enhancing the learning experience to create effective, efficient clinicians | [1] 1^st^, 2^nd^ & 3rd year Dietetics students  [2] 40 | [1] Single group  [2] Post-test design | [1] Not specified  [2] Controlled reflective processes underpinned the online workbook  NHS & HCPC placement standards | Knowledge, communication & professional practice domains | Mixed methods | [1] Not specified, but simulated patient journeys  [2] Statutory & mandatory training Virtual wards & mealtimes.  Recordings, peer learning with structured activities, & an online workbook  [3] 2 weeks | [1] 360 images  Diet-COMMS  COVCollaborate App  Microsoft Teams  [2] Screen based | Student evaluation  (questionnaire & focus group)  Web page metrics  Student results | 100% of the cohort passed the placement.  360 images:  Rated enjoyable & informative.  Were accessed 1016 times | Despite some concerns / issues, a virtual placement can be a useful, rich experience for the student. | Funding not mentioned. No conflicts of interest |
| [1] Villa et al. (2021)^50^  [2] USA  [3] Western Journal of Emergency Medicine | To create, implement & evaluate a virtual clerkship with a focus on social emergency medicine (EM) & profession-al develop-ment | [1] 4^th^ year Medical students on an EM Clerkship  [2] 26 | [1] Single group  [2] Repeated measures design | [1] Clerkship director, associate programme directors, medical education fellows and senior EM residents. Needs assessment of post graduate near peers.  [2] Kern's method for curriculum development  Framework for maximising online learning | Advanced medical  Knowledge  Social determinants of health  Professional development  Professional identity formation. | Mixed methods | [1] Paediatric anaphylaxis, motorcycle trauma, hypothermia & abdominal aortic aneurysm Themes: language, incarceration, gender identity, race & homelessness  [2] Assignments (using websites & podcasts), small group didactic sessions, student led teaching, virtual escape rooms & book club.  [3] 2 weeks | [1] Zoom  Foundations of EM (online resource)  IDHEAL modules  [2] Screen based | Pre & post knowledge tests  Survey evaluations:  Overall attitude to the course  After each module x 5  (to determine the comfort with applying content to a clinical setting) | 75% & 96% survey response rates: post-module & end-rotation  Modest gains in knowledge scores (p=0.006, effect size: 0.68, 95% CI 0.12-1.24)  89% strongly agreed: topics were important.  95% strongly agreed /agreed: rotation should be repeated  Positive feedback for course design, but zoom fatigue was mentioned. | A virtual EM visiting clerkship is feasible, supports knowledge acquisition & is perceived as valuable by participants.  Virtual learning  experiences may be valuable in the future as an adjunct to  traditional in-person rotations. | None |

| Citation:  Author  (year)^1^  Country^2^  Publication^2^ | Purpose / aim | Population:  Profession  (specialist rotation)^1^  Sample Size^2^ | Study Design:  Comparison group^1^  pre / post measures^2^ | VSP Design:  Stakeholders Involved  Pedagogical framework | Desired capabilities | Methodology | Intervention:  Scenario(s)^1^  Activities^2^  Duration^3^ | Delivery:  Software^1^  Hardware^2^ | Student focussed outcome measures | Key Findings | Conclusions/ Implications | Funding  / Conflicts of interest |
| --- | --- | --- | --- | --- | --- | --- | --- | --- | --- | --- | --- | --- |
| [1] Weston & Zauche (2021)^51^  [2] USA  [3] Nurse Educator | To compare the Assessment Technologies Institute (ATI) scores of students  who completed their practicum in person versus virtually | [1] 2^nd^ Semester prelicensure baccalaureate nursing students on a paediatric clinical course  [2] 186 | [1] In-person placement (clinic & simulation) comparator group (n=93)  [2] Post-test measures | [1] Not specified  [2] Not mentioned | Take a history  Perform physical assessment  Identify problems  Prioritise interventions  Integrate foundation knowledge | Quantitative | [1] Physical assessment, sickle cell, cystic fibrosis, infectious respiratory disease, head injury, cardiovascular disease  [2] Prebrief,  i-Human cases, debrief in groups & quizzes  [3] 5 weeks | [1] Online conferencing (not specified)  i-Human  [2] Screen based | Scores on the ATI exam | No difference between the scores on the ATI exam between groups t(184)=0.700 (p=0.485) | Using the  i-Human platform with prebriefing & debriefing, is an effective approach to simulating a pediatric clinical practice | Not stated |
| [1] White et al (2021)^52^  [2] USA  [3] Archives of Pathology & Laboratory Medicine | To develop & implement a digital slide–based virtual surgical pathology clinical elective in response to the temporary suspension of in person clinical rotations | [1] Medical students (Pathology rotation)  [2] 43 | [1]  Single group  [2] Post measures design | [1] Course director (and a needs assessment from student evaluations and rotation data over the proceeding 5 year period)  [2] Kern's 6 step approach to curriculum development | Summarise the role of a general surgical pathologist.  List the defining histologic features of several common pathologies.  Demonstrate how to determine the pathologic stage for an oncologic resection    Describe how to approach the assessment of a biopsy specimen | Quantitative | [1] Benign & Malignant neopasms. Non-neoplastic, developmental & inflammatory processes  [2] Reading (e-texts), e-lectures, virtual slides, quizzes, gross dissection videos, student led presentations  [3] 3 weeks | [1] Zoom PowerPoint Blackboard (LMS)  Inversus (Open EdX platform) Internal education web page: hosted the GI pathology module (designed using iSpring Suite 9) & videos.  Leica Aperio & Roche iScan (for slide digitisation). Concentriq (for slide delivery). Amazon Web Cloud storage.  [2] Screen based | Pass / fail assessment.  Student evaluation survey | All students passed the assessment.  39.5% survey response rate.  Learning objectives, patient variety, effective teaching / feedback, the value of technology & the quality of the educational experience were all highly rated | Provided a meaningful clinical experience in a time of online education need.  Added benefits included increased medical student exposure to pathology as a medical specialty & demonstration of how digital slides can potentially improve standardisation of the pathology clerkship. | None |

| Citation:  Author  (year)^1^  Country^2^  Publication^3^ | Purpose / aim | Population:  Profession  (specialist rotation)^1^  Sample Size^2^ | Study Design:  Comparison groups^1^  pre / post measures^2^ | VSP Design:  Stakeholders Involved^1^  Pedagogical Framework^2^ | Desired capabilities | Methodology | Intervention:  Scenario(s)^1^  Activities^2^  Duration^3^ | Delivery:  Software^1^  Hardware^2^ | Student focussed outcome measures | Key Findings | Conclusions/ Implications | Funding  / Conflicts of interest |
| --- | --- | --- | --- | --- | --- | --- | --- | --- | --- | --- | --- | --- |
| [1] Wik et al (2022)^53^  [2] Canada  [3] International Journal of Nursing Education Scholarship | To use a community health virtual simulation program to provide clinical placements for undergraduate students | [1] 2^nd^ year Nursing students (community rotation)  [2] 16 | [1] Single group  [2] Post-test design | [1] Not specified  [2] Not stated | Determining factors that impact community health.  Gaining insights about diverse health needs, community interventions | Qualitative | [1] Health, social & environmental issues. Infectious disease outbreaks, mental health & cyber bullying  [2] Observation Applying the community as partner model Windshield surveys Key informant interviews.  Planning, implementing & evaluating media campaigns  Home assessment Presentations  Infographic design Written reflection Quality improvement  [3] 16 weeks | [1] Sentinel City 3.1  Zoom  PowerPoint  [2] Screen based | All students submitted quality improvement recommendations& 3 of them co-authored a quality improvement assessment with the faculty | Overall, students felt that Sentinel City®3.1 was an adequate program for meeting course objectives.  The prescripted design limited the opportunity for critical thinking. | Overall, students who provided feedback considered the platform to  be a safe and effective way to teach community and population health nursing concepts and skills. | No funding or conflicts of interest |
| [1] Williams et al (2021)^54^  [2] USA  [3] Urology | To design, implement & evaluate learner attitudes of a virtual urologic surgery clinical rotation for medical students. | [1] Senior Medical students (Urology sub-internship)  [1] 10 | [1] Single group  [2] Repeated measures design | [1] Not specified  [2] Aligned with the Urological Association Medical Students Curriculum | Urologic evaluation  Case presentation  Anatomy & pathology of common conditions,  Literature appraisal | Mixed | [1] Benign, oncologic & paediatric urology  [2] lectures, problem-based learning, reading & videos, discussion board, videoconferences presentations & literature reviews  [3] 2 weeks | [1] Canvas (LMS)  BlueJeans (videoconferencing)  [2] Screen based | Comfort with performing urologic evaluations, confidence in knowledge, identifying conditions & placing consults for urologic issues | Significant (p<0.05) increases in: self-perceived knowledge, comfort with performing evaluations, confidence in naming conditions & placing consults | Virtual rotations are scalable & effective at delivering surgical material and can approximate the interpersonal teaching found in clinical learning environments. | Not stated |

| Citation  Author  (year)^1^  Country^2^  Publication^3^ | Purpose / aim | Population  Profession  (specialist rotation)^1^  Sample Size^2^ | Study Design  Comparison group^1^  pre / post measures^2^ | VSP Design  Stakeholders Involved  Pedagogical framework | Desired capabilities | Methodology | Intervention:  Scenario(s)^1^  Activities^2^  Duration^3^ | Delivery:  Software^1^  Hardware^2^ | Student focussed outcome measures | Key Findings | Conclusions/ Implications | Funding  / Conflicts of interest |
| --- | --- | --- | --- | --- | --- | --- | --- | --- | --- | --- | --- | --- |
| [1] Zhou et al. (2020)^55^  [2] China  [3] Telemedicine and e-health | To observe & analyse the application of Massive Open Online Course (MOOC) & micro video during the COVID-19 epidemic. | [1] Trainee nurses on an Emergency Department (ED) rotation  [2] 60 | [1] in-person (traditional) placement  comparator group (n=30)  [2] Post-test measures | [1] Nursing Skills Group, including a teaching supervisor, clinical nursing expert & a photographer  [2] Content was based on the syllabus of the Emergency & Critically Ill Nursing textbook | Theoretical & practical skills  with COVID-19 prevention & protection level strategies | Quantitative | [1] Operation of specialised nursing skills involved in the ED  [2] MOOC & Micro Video course  [3] 2 weeks | [1] The ED network training platform  MOOC (based on the textbook)  [2] Screen based | Theoretical & practical exam scores  Student evaluation | There was no significant difference in exam scores between groups  100% survey response rate  Overall satisfaction, degree of easy understanding, teacher evaluation & learning results group were higher in the experimental group, with statistical significance  (p < 0.05) | Combined mode of MOOC micro-video can present theoretical and practical courses in a unique way, & is  a better alternative when face-to-face and practical  courses can no longer be carried out. | None |
