# Supplementary material for: Virtual Simulated Placements in Health Care Education: Scoping Review
Source: JMIR Med Educ. 2025 Jun 10;11:e58794. doi: 10.2196/58794 (PMC12280114; doi:10.2196/58794)
Supplement: Multimedia Appendix 5 [file mededu-v11-e58794-s005.docx]

Appendix 6: – Papers by Professions

Breakdown by Nursing Specialty

Breakdown by Medical Specialty

| Paediatrics | 2 |
| --- | --- |
| Community | 1 |
| Emergency | 1 |
| Psychiatric | 1 |
| Multiple | 1 |
| None | 1 |

| Radiology | 4 |
| --- | --- |
| Emergency | 2 |
| Pathology | 2 |
| Anaesthesiology | 1 |
| GP | 1 |
| Internal Medicine | 2 |
| Neurology | 1 |
| Orthopaedics | 1 |
| Otolaryngology (ENT) | 1 |
| Urology | 1 |
| None | 1 |
| Multiple | 2 |

This is a Multimedia Appendix to a full manuscript published in the J Med Internet Res. For full copyright and citation information see http://dx.doi.org/10.2196/jmir.xxxx
