# Supplementary material for: Virtual Simulated Placements in Health Care Education: Scoping Review
Source: JMIR Med Educ. 2025 Jun 10;11:e58794. doi: 10.2196/58794 (PMC12280114; doi:10.2196/58794)
Supplement: Multimedia Appendix 6 [file mededu-v11-e58794-s006.docx]

Appendix 7: Conceptual Frameworks

This is a Multimedia Appendix to a full manuscript published in the J Med Internet Res. For full copyright and citation information see http://dx.doi.org/10.2196/jmir.xxxx

| Underpinning Concepts | Type of concept | Papers |
| --- | --- | --- |
|  |  |  |
| Pedagogy | Student centred learning | Luo et al. (2021)^41^ |
|  | Andragogy | Bhaysham and Dyer (2020)^29^  Creagh et al. (2021)^30^ |
|  | Problem based learning | Bhaysham and Dyer (2020)^29^  Creagh et al. (2021)^30^  Kasai et al. (2021)^39^ |
|  | Experiential learning | Creagh et al. (2021)^30^ |
|  | Reflective practices | Taylor et al. (2021)^49^ |
|  | Online learning | Fehl et al. (2022)^33^  Villa et al. (2021)^50^ |
|  |  |  |
| Theoretical Frameworks | VSP development | Ganji et al. (2022)^34^ |
|  | Curriculum development | Nguyen et al. (2023)^43^  Redinger and Greene (2021)^45^  Samueli et al. (2020)^46^  Villa et al. (2021)^50^  White et al. (2021)^52^ |
|  |  |  |
| Standards or  Existing curricula | International Nursing Association for clinical simulation and learning (USA) | Kubin et al. (2021)^40^ |
|  | National Standards for Nursing Undergraduates (China) | Luo et al. (2021)^41^ |
|  | NHS and HCPC Placement standards (UK) | Taylor et al. (2021)^49^ |
|  | Urological Association of Medical Students Curriculum (USA) | Williams et al. (2021)^54^ |
|  | Syllabus of the Emergency and Critically Ill Nursing Textbook (China) | Zhou et al. (2020)^55^ |
