# Supplementary material for: Virtual Simulated Placements in Health Care Education: Scoping Review
Source: JMIR Med Educ. 2025 Jun 10;11:e58794. doi: 10.2196/58794 (PMC12280114; doi:10.2196/58794)
Supplement: Multimedia Appendix 7 [file mededu-v11-e58794-s007.docx]

Appendix 8: Bespoke Healthcare Technology

| Type of Resource | Name of Resource | Papers |
| --- | --- | --- |
| Commercial Software  (Virtual cases) | Aquifer | Creagh et al. (2021)^30^  Durfee et al. (2020)^32^ |
|  | Body Interact | De Ponti et al. (2020)^31^ |
|  | vSim | Joung and Kang (2022)^38^  Kubin et al. (2021)^40^  Luo et al. (2021)^41^ |
|  | NurseThink vClinical | Kubin et al. (2021)^40^ |
|  | F.A. Davis Paediatric Interactive Clinical Scenarios for RNs | Kubin et al. (2021)^40^ |
|  | i-Human | Weston and Zauche (2021)^51^ |
|  | Sentinel City | Wik et al. (2022)^53^ |
| Online modules/training | Cleveland Clinic Paediatric Radiology Modules | Gomez et al. (2020)^35^ |
|  | Virtual Healthcare Experience | Kubin et al. (2021)^40^ |
|  | National Prescribing Module | Smith and Jones (2023)^47^ |
|  | Online MedEd CaseX | Redinger and Greene (2021)^45^ |
|  | Emergency Medicine Reviews and Perspectives | Redinger and Greene (2021)^45^ |
|  | Diet-COMMS | Taylor et al. (2021)^49^ |
|  | IDHEAL Modules | Villa et al. (2021)^50^ |
|  | Foundations of EM |  |
|  | Scientific Foundations of Medicine (SFM) histology and pathobiology | White et al. (2021)^52^ |
|  | Gastrointestinal (GI) Pathology |  |
| Custom built sites/apps |  |  |
|  | Internal trainee education page | White et al. (2021)^52^ |
|  | Google Sites | Kubin et al. (2021)^40^ |
|  | Articulate.com | Rahm et al. (2021)^44^ |
|  | 360 images | Taylor et al. (2021)^49^ |
|  | The ED Network Training Platform | Zhou et al. (2021)^55^ |
| Online resources | Pacsbin  TeamRads.com  LearningRadiology.com  CTisUs.com  High Value Practice Academic Alliance Ordering Wisely E-Lectures | Gomez et al. (2020)^35^ |
|  | One Night in the ED | Gomez et al. (2020)^35^  Redinger and Greene (2021)^45^ |
|  | Sublux Radiology | Redinger and Greene (2021)^45^ |
|  | John Hopkins Surgical Pathology (login required) | White et al. (2021)^52^ |
| Whole slide image viewers | Concentriq |  |
|  | CaseViewer  Aperio ImageScope | Samueli et al. (2020)^46^ |

This is a Multimedia Appendix to a full manuscript published in the J Med Internet Res. For full copyright and citation information see http://dx.doi.org/10.2196/jmir.xxxx
